# Metagenomic insights of the infant microbiome community structure and function across multiple sites in the United States

**DOI:** 10.1038/s41598-020-80583-9

**Published:** 2021-01-21

**Authors:** Giorgio Casaburi, Rebbeca M. Duar, Heather Brown, Ryan D. Mitchell, Sufyan Kazi, Stephanie Chew, Orla Cagney, Robin L. Flannery, Karl G. Sylvester, Steven A. Frese, Bethany M. Henrick, Samara L. Freeman

**Affiliations:** 1 Evolve BioSystems, Inc., Davis, CA 95618 USA; 2 Department of Surgery, Stanford University, Stanford, CA USA; 3 Department of Food Science and Technology, University of Nebraska, Lincoln, NE 68588 USA; 4 Present Address: Department of Nutrition, University of Nevada, Reno, Reno, NV 89557 USA

**Keywords:** Metagenomics, Microbiome

## Abstract

The gut microbiome plays an important role in early life, protecting newborns from enteric pathogens, promoting immune system development and providing key functions to the infant host. Currently, there are limited data to broadly assess the status of the US healthy infant gut microbiome. To address this gap, we performed a multi-state metagenomic survey and found high levels of bacteria associated with enteric inflammation (e.g. *Escherichia*,* Klebsiella),* antibiotic resistance genes, and signatures of dysbiosis, independent of location, age, and diet. *Bifidobacterium* were less abundant than generally expected and the species identified, including *B. breve, B. longum* and *B. bifidum,* had limited genetic capacity to metabolize human milk oligosaccharides (HMOs), while *B. infantis* strains with a complete capacity for HMOs utilization were found to be exceptionally rare. Considering microbiome composition and functional capacity, this survey revealed a previously unappreciated dysbiosis that is widespread in the contemporary US infant gut microbiome.

## Introduction

The neonatal period represents a unique stage of life when critical foundations of lifelong health are established^1,2^. Throughout this period, the gut microbiome can provide protection against enteric infections and is crucial for the proper development of the immune system^3,4^. In infants, it is well established that gut microbiome perturbations characterized by the overrepresentation of potentially pathogenic taxa are implicated in mediating persistent pathophysiological and immune abnormalities, including heightened risk for immunological disorders later in life and acute chronic inflammation^3,5–7^. These gut microbial perturbations, conceptually referred to as dysbiosis, are strongly associated with the absence of infant-associated *Bifidobacterium* that encode key functions required for the metabolization of human milk oligosaccharides (HMOs) in the infant^8^.

Disruptions to the optimum infant intestinal microbiome are thought to result from changes to the infant’s diet, antibiotic exposure, and cesarean section delivery, as well as other interventions or exposures that might alter the transmission of microbes from mother to infant or the communities themselves^9–12^. However, there is mounting evidence suggesting that disparities between microbiome compositions observed today in different human populations may also echo historical changes including migrations^13^, historical infant feeding practices^14^, or the sum of all changes associated with modern lifestyles^15^. Despite the importance of the microbiome for infant health, nutrition, and development, most reports on the infant gut microbiome in developed countries have described communities in preterm infants^16,17^, and only few studies have utilized metagenomics to characterize microbiome function at distinct sites from large cohorts of infants^18,19^. Recent metagenomic surveys focused on genetically similar, but socioeconomically distinct populations in three European countries identifying microbiome compositions in early life with clear implications for long term health and development regarding the development of autoimmune diseases including Type 1 Diabetes^19^. Other studies have identified health status-specific differences among infants in low-income countries that later impacted vaccine responses, further signaling the important role that the microbiome in early life plays in immune development^20,21^. Similarly, recent large-scale studies have identified parallel differences in the microbiomes among infants even within high-income countries in Europe, which were associated with the development of autoimmune diseases such as asthma^22^. Mechanistic support for the critical role *Bifidobacterium* play in early life immune development has also been recently described^23^. Thus, it appears increasingly important to describe population-level norms among otherwise healthy infants within and across countries, especially large, ethnically, and socioeconomically diverse countries such as the United States.

In order to begin to address this need for population level surveys of the healthy infant gut microbiome in the United States, fecal samples from 227 infants (0–6 months of age) were collected from pediatric research sites located in five states, representing a cross-section of infants from across the United States. Stool samples underwent shotgun metagenomics sequencing. Specifically, we applied shotgun metagenomics to characterize: (1) gut bacterial communities of healthy US infants in the first 6 months of life; (2) ecosystem functions by determining the metabolic potential of gut microbiomes in different enterotypes to metabolize human milk oligosaccharides (HMOs) from breast milk; and (3) the carriage of antibiotic resistant genes (ARGs) in infants across different US states. The above criteria were then used to classify microbiomes as dysbiotic or not, based on the concept of ecosystem services^24^ adapted to evaluate the benefits infants receive from functions provided by their gut microbiomes^8^.

Overall, this survey revealed that, on average, infants in the US have a low abundance of *Bifidobacterium*, a high abundance of potentially pathogenic bacteria carrying high levels of ARGs, as well as limited capacity of metabolizing HMOs from breast milk. These findings were widespread in infants in the US, independent of location, age, and diet. This survey offers a new perspective when considering infants in the context of a healthy microbiome and the acute and long-term consequences it implies.

## Results

### Infant gut microbiome compositions vary independently of age, diet and location

**Figure 1:**
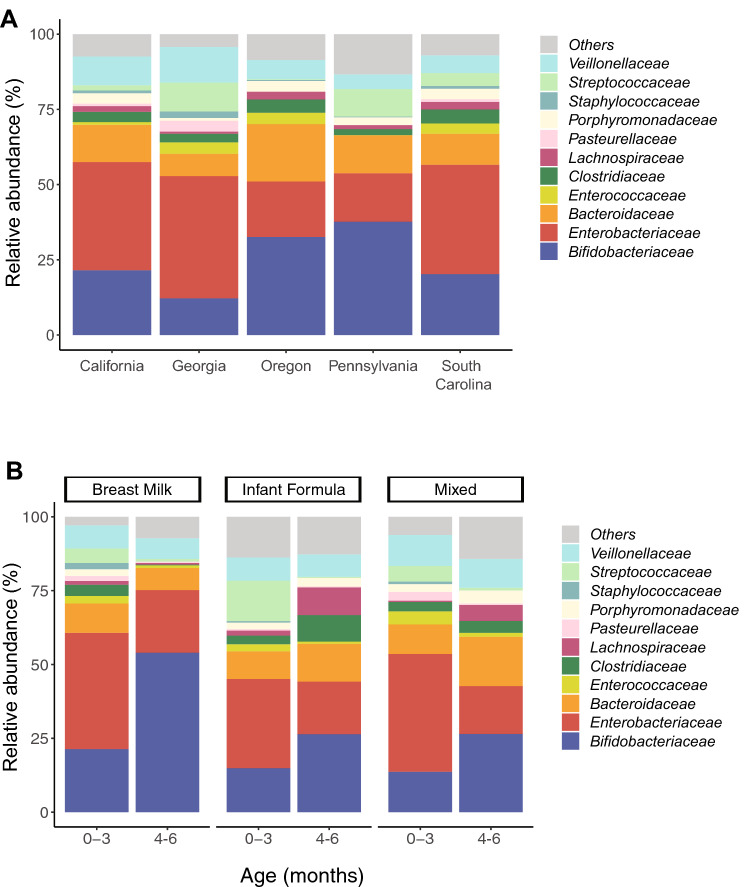
Relative abundance of bacterial families by location, diet and age. (**A**) Relative abundance (%) of top bacterial families identified in infants 0–6 months of age across five US states. (**B**) Relative abundance (%) of top bacterial families identified and grouped by age and diet.

Our cohort of 227 samples (one sample per subject) from 7 research sites in 5 states (California, Georgia, Oregon, Pennsylvania, South Carolina), was assessed for microbiome composition and function via shotgun metagenomic sequencing. Demographic data for every subject is reported in Supplementary Table 1. After quality filtering, Illumina sequencing led to an average of 28 million PE reads per sample (± SD 6.4 million), of which 4.6% (± SD 12%) per sample on average were discarded as human reads. High-quality, human-filtered reads were subjected to taxonomic and functional profiling (see “Methods”). A total of 367 classified bacterial species belonging to 119 genera, 53 families, 25 orders, 15 classes, and 7 phyla were identified across the samples (Supplementary Table 2). Potential pathogenic bacteria were present among the top ten bacterial families, which combined accounted for 93% of the infant’s microbiome. The most abundant on average were *Enterobacteriaceae* (35%), *Streptococcaceae* (5.6%), *Clostridiaceae* (3.6%), and *Staphylococcaceae* (1.23%) (Fig. 1A). The bacterial family of *Bifidobacteriaceae* was low on average in infants from all five states (19.9%) with the lowest abundance in infants from Georgia (12%) and the highest (37%) in Pennsylvania. When considering only samples collected within the first 100 days of life (0–3 months), a critical window of time for immune system development^25^, we did not see a difference by state in terms of bacterial families composing the microbiome, with a composition that appears to be independent of location^25^ (Supplementary Fig. 1). Splitting the data by both age (0–3 vs. 4–6 months) and diet (exclusively breast or formula fed and mixed), revealed that the 0–3 months samples had a higher individual variation in bacterial family composition compared to the 4–6 months samples, probably due to the natural acquisition of bifidobacteria (Fig. 1B).

Furthermore, we performed a microbiome multivariable association analysis with linear models, using MaAsLin2 (see “Methods”), by including all the clinical variables available in this study to test for microbiome differences at the family level according to metadata. Interestingly, while we did find significant taxa associated with few metadata variables, most of the bacterial families reported, were very low in relative abundance overall (< 2%) and thus we do not believe that their biological impact is of a major concern or clinical interest. However, two top families, namely *Bifidobacteriaceae* (19.9%) and *Bacteroidaceae* (10.4%) were all significantly associated with age but not with any other variable (Supplementary Table 6), confirming again the natural acquisition of these families from the environment as the infants grow older.

At the species level, we identified known potentially pathogenic species of particular relevance, especially as it pertains to the neonatal population, accounting for 26% of the microbiome on average in all samples (Supplementary Table 2). In order of abundance we identified, *Escherichia coli* (12.6%) the most abundant among all species overall, *Klebsiella pneumoniae* (7%), *Klebsiella oxytoca* (2.5%), *Enterobacter cloaceae* (2.7%), *Clostridium perfringens* (1.1%), *Clostridium difficile* (0.1%), *Staphylococcus aureus* (0.03%) and *Streptococcus agalactiae* (0.01%). We also identified a taxon classified as *Escherichia* (5%) for which the species level assignment was not classified.

### Bifidobacteria levels are low on average in the US independent of diet

**Figure 2:**
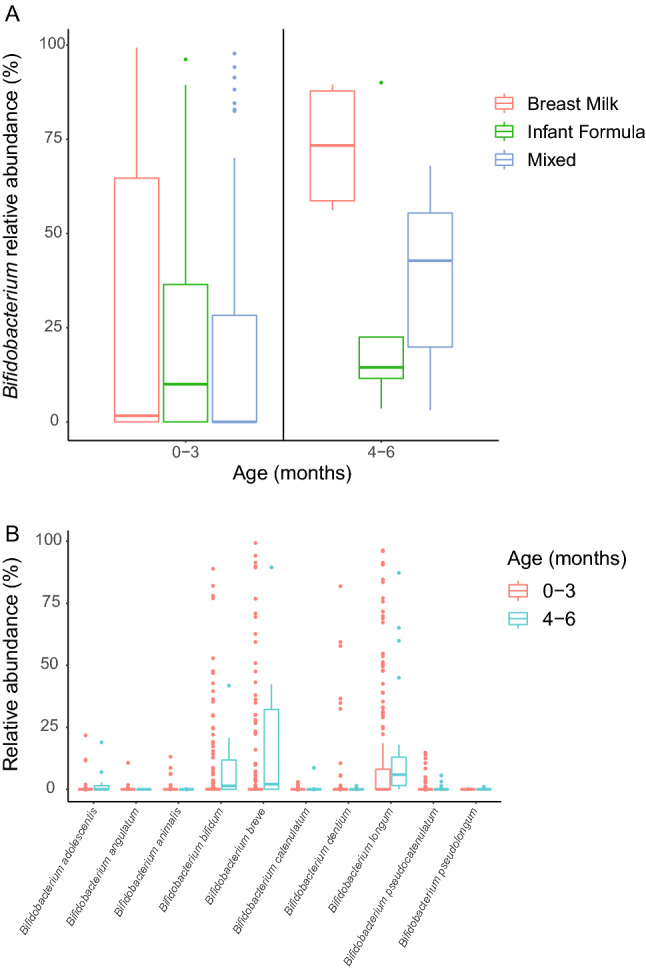
Relative abundance of *Bifidobacterium* at the genus and species level. (**A**) *Bifidobacterium* relative abundance (%) grouped by age and diet. (**B**) Relative abundance (%) of all species identified within the *Bifidobacterium* genus and grouped by patient age. The lower and upper hinges of the box plots correspond to the first and third quartiles (the 25th and 75th percentiles), while internal hinge is the 50th percentile (median). Data beyond the end of the whiskers are "outlying" points and are plotted individually.

As bifidobacteria are considered a fundamental component of a healthy infant gut microbiome, we performed targeted genus-, species- and strain-level metagenomic analysis using unique clade-specific marker genes (see “Methods”). Overall, the average abundance of *Bifidobacterium* was 20%. When accounting for diet, within the first 100 days of life (0–3 months) or later (4–6 months), there were no significant differences in the abundance of *Bifidobacterium,* independent of whether the infants were exclusively fed breast milk, formula or were fed a mixed diet of breast milk and formula (Fig. 2A). However, breastfed infants in the 4–6 months age group, had a higher abundance of *Bifidobacterium* (75% of the microbiome on average), though not significantly different (*P* > 0.05) amongst feeding types, likely due to high individual variation from the formula fed infants in that age group, which had an average abundance of *Bifidobacterium* below 25%. At the species level, we identified 10 *Bifidobacterium* species, with the most abundant being *Bifidobacterium longum* (8.5%), *Bifidobacterium breve* (5.8%), and *Bifidobacterium bifidum* (3.75%) (Fig. 2B)*.* All three species were significantly lower in the 0–3 months age group compared to the older infants (4–6 months) and had a greater degree of intra-group variability with samples ranging from 0% to more than 90% of relative abundance of *B. breve, B. longum,* or *B. bifidum*.

### *B. infantis*, a key infant gut symbiont, is missing in 90% of infants

We assessed the bifidobacteria composition at the strain level by investigating the presence of genes specifically involved in the consumption of human milk oligosaccharides (HMOs)^26,27^. We performed a nucleotide-level analysis of six gene clusters previously identified to be relatively unique to the genome of *Bifidobacterium longum* subsp. *infantis* (*B. infantis*)^27^. Notably, the 43 kb H1 gene cluster that encodes glycosyl hydrolases active on four key HMO glycosidic linkages, has so far only been identified in *B. infantis*^26^ and is broadly conserved among the subspecies^8,28^ while other HMO-related gene loci are less conserved and found among different bacteria beyond bifidobacteria^27^. To account for genomic variation within the subspecies, we used the detection of at least 90% of the genes in these six genomic loci (H1, H2, H3, H4, H5, and a urease gene cluster) to detect *B. infantis* among infant samples by metagenomic sequencing. Using this criterion, we detected *B. infantis* in only 10% (23 samples) of all 227 samples. However, of these samples, only 3% (6 samples) had a relative abundance of *B. longum* species (inclusive of *B. infantis*) greater than 40%, which collectively represented a mean abundance of 65.10% (± SD 19.74%). Among the remaining 17 samples (7% of samples) where *B. infantis* was detected, the overall *B. longum* species only achieved a mean relative abundance of 7.38% (± SD 6.95%).

To confirm our findings, we also applied a subspecies-specific qPCR assay (see “Methods”) to detect *B. infantis* among these samples. Using qPCR, we detected *B. infantis* in 11% of samples (*n* = 24), in strong agreement with our metagenomic detection approach (Supplementary Table 1). One sample not detected by our metagenomics approach, but positive by qPCR, demonstrated a low relative abundance of *B. longum* species (%) and was missing more than 33% and 42% of the genes in the H1 and urease gene clusters, respectively.

**Figure 3:**
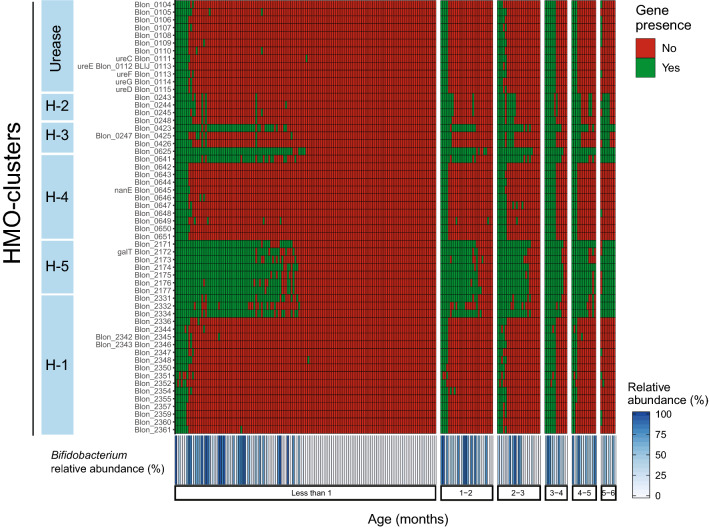
Functional HMO-cluster gene profiling by samples. (**A**) Heatmap showing presence/absence of the 56 HMO-cluster genes in all samples in the survey. Every column represents an individual sample and ordered by age block. Within a age block samples are ordered by decreasing presence of genes from left to right. Bottom of heatmap shows relative abundance (%) of *Bifidobacterium* by corresponding individual samples.

Thus, we used the detection of the H1 gene cluster as a proxy to determine the presence of *B. infantis* in the gut microbiome. All the genes belonging to the H1-cluster were detected in only 7 out of the 227 microbiome samples analyzed, meaning 97% of infants were likely missing *B. infantis*. Additionally, all 220 infants with incomplete or missing genes in the H1-cluster were also lacking crucial genes from other H-clusters non uniquely found in *B. infantis*, which are also involved in the capture, transport and metabolism of HMOs^27^ (Fig. 3). Furthermore, a comparison of the relative abundance of the genus *Bifidobacterium* on a per sample basis against the presence of the H-clusters showed that even with high abundance of *Bifidobacterium* (> 90%) in some of the infant microbiomes, key HMO genes were largely undetected (Fig. 3). This suggests that even when high levels of *B. breve, B. longum,* or *B. bifidum* are found in infants, it does not translate into a complete functional capacity of HMO utilization. Together, these analyses revealed that genes crucial to the utilization of HMOs, and therefore the main species that encodes them (i.e., *B. infantis*), are largely absent from the microbiome of infants in the US, independent of age, location, and diet (Fig. 3).

### Antibiotic resistance in the US infant microbiome

**Figure 4:**
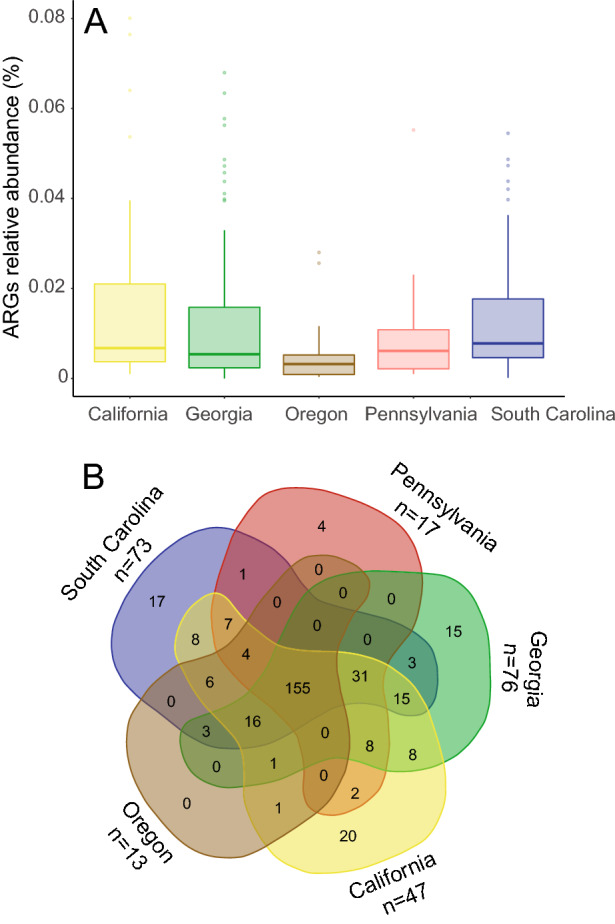
Antibiotic resistance genes characterization by state. (**A**) Relative abundance (%) of antibiotic resistance genes (ARGs) compared to overall microbiome composition identified by state. (**B**) Venn diagram showing shared ARG types by state. 155 ARGs types were core to all states (center of diagram), with unique ARG types identified in single state reported at the edge of the diagram. *N*’s represent number of patient samples by state.

Analysis of the total load of antibiotic resistance genes (ARGs) in the infants’ microbiomes across the US (i.e., the resistome), revealed a high abundance of ARGs in all five states analyzed in this survey, with a total of 325 unique ARGs identified from the CARD database (Supplementary Table 3). Particularly, South Carolina, California and Georgia had the highest abundance of ARGs (0.10%/0.014%/0.013%, average ARGs relative abundance compared to overall microbiome), followed by Pennsylvania (0.01%) and Oregon (0.006%) (Fig. 4A). No significant differences were observed by state in terms of overall ARG % microbiome composition. A total of 155 ARGs were common to the five states and despite the total abundance levels, more unique ARGs were identified in California (*n* = 20) (Fig. 4B). After accounting for sequencing depth, we confirmed that both the type and load of ARGs identified were not related to the number of sequences per sample grouped by state (Oregon = 32.5 M; Georgia = 28.9 M; California = 28.1 M; Pennsylvania = 27.5 M; South Carolina = 26.2 M), but rather possibly related to the number of individual patient samples collected (Fig. 4B). However, there appears to be a virtual plateau in ARG diversity assessed in this study considering that California had the highest diversity with almost half the patient samples compared to Georgia. A complete list of the identified ARGs by state is reported in Supplementary Table 4. Interestingly, the 20 unique ARGs found in California in this study have been also previously found in another cohort of 60 healthy, exclusively breastfed infants lacking *B. infantis* at day 21 of life^29^. Amongst the core ARGs found in every state, the most abundant on average were “ARO_3003369” (*Escherichia coli* EF-Tu mutants conferring resistance to Pulvomycin); followed by “ARO:3003317” (*Salmonella serovars* parE conferring resistance to fluoroquinolones); and “ARO:3003890” (*Escherichia coli* UhpT with mutation conferring resistance to fosfomycin). When considering age, there was a higher load of ARGs in the microbiome of infants between 0 and 3 months, though due to the high degree of intra-individual variation, not significantly different from the 4–6 months age window (*P* > 0.05) (Supplementary Fig. 2). We analyzed the ARG abundance on both the gene type and antimicrobial class levels using principal coordinate analyses (PCoA). With regard to the sample resistome dissimilarities at both genes and drug class levels, there was no clear separation when considering all the metadata collected, though geography and age were significant, but these factors had a very low effect size (adonis) on the overall resistome (Supplementary Table 5). Collapsing the ARGs into their respective drug class and by state, we found that on average 54% of the resistome was associated with genes conferring multi drug resistance, followed by fosfomycin, elfamycin and fluoroquinolone antibiotics resistance (Supplementary Fig. 3).

### Enterotypes of the US infant gut microbiome and functional analysis of HMO-metabolism potential

**Figure 5:**
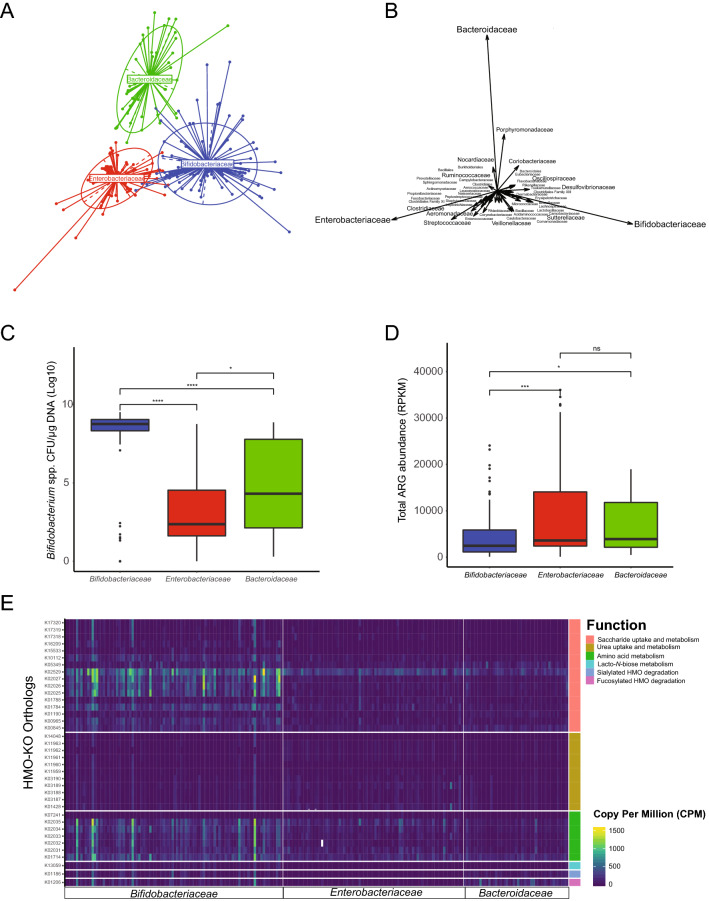
Enterotype grouping, *Bifidobacterium* abundance and functional gene annotation. (**A**) Enterotype analysis of samples at the family level. Samples clustered in three different enterotypes reported by different colors. In the center of the cluster is shown the main bacterial family responsible for the enterotype formation. (**B**) Bacterial families driving enterotype formation. Longer arrows indicate a stronger contribution. (**C**) Quantification of total *Bifidobacterium *spp. by enterotype. (**D**) Abundance of ARGs in all samples grouped by enterotype. (**E**) Heatmap showing relative abundance and functional annotation of KEGG orthologous genes related to HMO-utilization across enterotypes.

Three enterotype clusters were identified; 99 subjects were assigned to *Bifidobacteriaceae*, 81 to *Enterobacteriaceae*, and 47 to *Bacteroidaceae* enterotype (Fig. 5A). Other bacterial taxa identified contributed less significantly to each enterotype cluster compared to the three main bacterial families highlighted (Fig. 5B). Relative abundances of the three bacterial taxa that are principally responsible for the separation of samples confirmed expected predominance of *Bifidobacteriaceae*, *Enterobacteriaceae* and *Bacteroidaceae*, respectively (Supplementary Fig. 4). We confirmed the enterotype classification results using other well-known clustering approaches, including principal component analysis (PAM) as well as Bray–Curtis, which are commonly used to compare microbiome compositions across a given variable; here, our enterotype cluster assignments. We used the permutational multivariate analysis of variance (adonis) to test whether the clustering by our Enterotype classification was supported. The PAM clustering reported a *P* = 0.001; R^2^ = 0.17, while Bray–Curtis reported a *P* = 0.001; R^2^ = 0.32, confirming that the Enterotypes are indeed different in terms of the composition and represent distinct compositional states. Furthermore, absolute abundances as measured via quantitative PCR targeting the *Bifidobacterium* genus, also confirmed a significantly higher abundance of *Bifidobacterium* within the *Bifidobacteriaceae*-driven enterotype compared to the *Enterobacteriaceae* (*P* < 0.0001, Holm-adjusted Dunn’s) or *Bacteroidaceae* (*P* < 0.0001, Holm-adjusted Dunn’s). Subjects in each enterotype did not significantly differ according to diet, location, or sex. However, when considering age, there was a significant difference between the *Bifidobacteriaceae* and *Enterobacteriaceae* enterotypes (*P* < 0.0001, Bonferroni).

Analysis of the resistome composition measured by the total amount of ARGs identified amongst enterotypes, showed a significant difference in reductions of the total abundance of ARGs in the samples clustering within the *Bifidobacteriaceae* enterotype compared to *Enterobacteriaceae* (*P* = 0.0004; Bonferroni) and *Bacteroidaceae* (*P* = 0.006; Bonferroni) enterotypes. No significant differences in the abundance of ARGs were observed between the *Enterobacteriaceae* and *Bacteroidaceae* enterotypes (*P* = 0.3; Bonferroni).

To determine if the enterotype classification was related to the HMO-metabolism potential in the microbiomes at the functional level, we determined the abundance of ortholog genes (KOs) involved in the uptake, degradation and metabolism of HMO in each of the enterotypes. We found that, on average, microbiomes belonging to the *Bifidobacteriaceae* enterotype had a significantly increased capacity for utilization of HMO (119 CPM, mean) compared to the *Bacteroidaceae* (43 CPM, mean; *P* < 0.0001; Holm-adjusted Dunn’s) and *Enterobacteriaceae* enterotypes (47 CPM, mean; *P* < 0.0001; Holm-adjusted Dunn’s) enterotypes. The HMO-metabolism potential was also significantly different between *Bacteroidaceae* and *Enterobacteriaceae* enterotypes (*P* = 0.04; Holm-adjusted Dunn’s). When grouping KOs by function, on average, those related to the uptake and degradation of sialylated HMOs were significantly more abundant in the *Bifidobacteriaceae* (69.7 CMP, mean) and the *Bacteroidaceae* (51.4 CMP, mean) enterotype compared to the *Enterobacteriaceae* (1.23 CMP, mean) enterotypes (*P* < 0.0001 and *P* < 0.0001, respectively), but not significantly different between the *Bifidobacteriaceae* and *Bacteroidaceae* enterotypes (*P* = 0.32; Wilcoxon-FDR). Functions related to lacto-*N*-biose metabolism were significantly different in all three enterotypes with the *Bifidobacteriacea* enteroype encoding the most functions (50.3 CMP, mean) and the *Enterobacteriaceae* enterotype encoding the least (0.62 CMP, mean). The ability to degrade fucosylated HMO was also differentially significant between enterotypes but was higher in the *Bacteroidaceae* enterotypes (159.9 CMP, mean), followed by the *Bifidobacteriacea* (97.7 CMP, mean) and the *Enterobacteriaceae* (13.8 CMP, mean). Orthologous genes encoding functions related to the transport and catabolism of mono and polysaccharides were not significantly different between the *Bacteroidaceae* (49.5 CPM, mean) and *Enterobacteriaceae* (52.0 CPM, mean) enterotypes (*P* = 0.48; Wilcoxon-FDR), but were significantly higher in the *Bifidobacteriaceae* enteroype (162 CPM, mean) compared to the *Bacteroidaceae* (*P* < 0.0001; Wilcoxon-FDR) and the *Enterobacteriaceae* (*P* < 0.0001; Wilcoxon-FDR) enterotypes. Urea-related KOs were not statistically different between *Bifidobacteriaceae* (26.1 CPM, mean) and *Enterobacteriaceae* (28.6 CPM, mean) enterotypes (*P* = 0.84; Wilcoxon-FDR), but were significantly lower in the *Bacteroidaceae* (13.5 CPM, mean) compared to the *Bifidobacteriaceae* (*P* < 0.0001; Wilcoxon-FDR) and the *Enterobacteriaceae* (*P* < 0.0001; Wilcoxon-FDR) enterotypes (Fig. 5). Finally, we determined the number of samples that had a complete set of HMO-utilization orthologous genes. We found that only 16 out of 227 had the complete set of these KO functions. Amongst them, more than half (*n* = 9) where classified in the *Bifidobacteriaceae* enterotype, while 5 were in *Bacteroidaceae* and 2 in the *Enterobacteriaceae* enterotype. By location, 5 infants were in California, 4 in Oregon, and 7 in South Carolina. By age, 5 infants were in the 4–6-month age block and the rest were 3 months or less. Individual family microbiome composition of the top bacterial families (≥ 1% relative abundance, on average), and sorted by enterotype classification, is also reported in Supplementary Fig. 5.

## Discussion

In the past few years, reliable evidence has emerged on the status of the US infant gut microbiome showing a general trend toward dysbiosis and associated negative acute- and long-term health consequences^23,30–32^. However, to date, researchers have derived conclusions mainly from single-site microbiome studies, which are often limited to a geographical area where samples were collected, or small association studies that carry inherent limits in terms of reproducibility of methodologies from sample collection to analysis. Furthermore, most infant microbiome studies in the US have focused on preterm infants, who are a very distinct population, experiencing epithelial gut immaturity and with a high degree of microbiome instability and a more severe dysbiosis compared to what is observed in term infants^31,33^.

Here, we aimed to broadly sample infants in the US across multiple geographies, in order to establish an understanding of the current status of the infant gut microbiome. Even though only five states were analyzed, our results suggest strong similarities across the research site regions, which appears to be independent of location, age and feeding modality (Fig. 1). Predominantly, 10 bacterial families accounted for 93% of the overall microbiome, while 40 families accounted for the remaining 7% on average. Amongst the 10 most abundant families, the majority were composed of potentially pathogenic species. Of the most abundant, the highest overall was *E. coli* (12.6% mean abundance), followed by *Klebsiella pneumoniae* (7% mean abundance). While the pathogenicity of these taxa is not universal^34,35^, in infants, the overrepresentation of γ-Proteobacteria, which includes the genera *Escherichia* and *Klebsiella*, are implicated in mediating pro-inflammatory responses associated with negative pediatric health outcomes^23,36,37^.

Several other bacterial species implicated with negative pediatric health outcomes, including sepsis and necrotizing enterocolitis (NEC) were identified^38,39^. For example, we detected relatively high levels of *Klebsiella oxytoca* (2.5% mean abundance) and *Enterobacter cloaceae* (2.7% mean abundance), both well described species related to NEC and *Streptococcus agalactiae* (Group B Strep; 0.01% mean abundance), which remains the primary cause of neonatal sepsis^40,41^. Infant microbiomes from across the US, irrespective of location, diet, or age could be classified into three distinct enterotypes, driven by abundances of *Bifidobacteriaceae* (43.6% mean abundance, *n* = 99), *Enterobacteriaceae* (35.7% mean abundance, *n* = 81)*,* or *Bacteroidaceae* (20.7% mean abundance, *n* = 47). Infants classified as *Enterobacteriaceae* enterotype were typified by the highest relative abundance of *Enterobacteriaceae,* and the highest abundance of antibiotic resistance genes (ARGs) (Fig. 5D). We detected a high amount of ARGs in a small number of species, with more than half of the genes associated with multi-drug resistance (Supplementary Fig. 3). Infants classified within the *Bifidobacteriaceae* enterotype had a lower abundance of pathogenic bacteria and ARGs compared to the infants in the other two enterotypes. These results are in accordance with previous reports showing microbiomes rich in *Bifidobacterium* carry fewer ARGs^29,42^ and have a lower abundance of *Enterobacteriaceae*. The antagonistic effects exerted by bifidobacteria against *Enterobacteriaceae,* which are known to carry a wide array of ARGs, may contribute to this association^43–45^. Interestingly, we did find specific ARG signatures by state. Particularly, 20 ARGs were found in this study to be uniquely present in Southern California and were the same genes previously reported in a Northern California infant microbiome study^46^. This suggests that there might be distinct resistomes by different geographical area, which may coincide with regional disparities in antibiotic use and stewardship^47^ and may warrant further investigation.

The delivery of ecosystem services in the infant gut such as impeding colonization of infectious bacteria are dependent on the efficient utilization of human milk oligosaccharides (HMOs) for the production of organic acids^8,44^. As HMO consumption is pivotal to the establishment of a bifidobacteria-rich microbiota in breastfed infants, the abundance of genes related to the uptake of HMO correlates strongly with the abundance of *Bifidobacterium* in infants from different nations, including the US^48^. Therefore, we screened our data for the presence of HMO-related orthologous genes known to be fundamental for the complete metabolism of HMOs^27^. The analysis of such orthologous (HMO-KO) by enterotype revealed that there was a significantly higher presence of these crucial genes in the *Bifidobacteriaceae-*driven enterotype compared to the other enterotypes. However, we found that although 43.6% of infants (*n *= 99) were classified as having a *Bifidobacteriaceae-*driven enterotype, the microbiomes of only a small subset (*n* = 16) of infants carried all the KO-HMO functions. Notably, HMO assimilation abilities significantly vary among different species and strains of *Bifidobacterium*^48,49^. Therefore, it is likely that species within the *Bifidobacteriaceae-*enterotypes were limited in the ability to access HMOs. Some *Bifidobacterium* (e.g., *B. bifidum*^50^) and *Bacteroides* possess enzymes for the purpose of degrading mucin glycans that hold structural similarities with HMO^51,52^. This may explain why functions related to sialic acid, which decorates both mucin and HMOs, were not differentially abundant between the *Bifidobacteriaceae* and *Bacteroidaceae* enterotypes. This idea is further supported by the fact that only 10% of the samples (*n* = 23) showed detectable *B. infantis,* which is the only known species fully equipped and ecologically adept at capturing, transporting and metabolizing HMOs^26^. Further, only a small number of these samples where *B. infantis* was detected (3% of total samples, *n* = 6) exhibited a microbiome composition characterized by a high abundance of *B. longum* species (inclusive of *B. infantis*) demonstrative of the efficient provision of ecosystem services^8^.

The absence or substantial decrease of *B. infantis* during the first trimester postnatal has been associated with (1) higher susceptibility to invasion of allochthonous bacteria with pathogenic potential^31,44^; (2) higher levels of ARGs^29^ and virulence factors^53^; (3) increased enteric inflammation^23^; (4) progressive mucus erosion compromising the intestinal mucosal barrier ^54^; (5) limited conversion of HMOs into organic acids^31^; (6) limited HMO-derived nutritional absorption^8^; and (7) altered response to vaccines^21^. Together, data generated in this study demonstrate that *B. infantis,* which is considered a key infant gut symbiont that has co-evolved with the host to provide important ecosystem services, is exceptionally rare among infants in the US^8,26,27,55^.

Our study carries the limitations of a cross-sectional study and infants analyzed were mostly located in urban and suburban areas and may not be representative of rural populations^56,57^. Also, the use of medications (e.g. antibiotics) and delivery mode are known to alter the microbiome^58,59^, but the impact of these interventions was not examined in the context of this study. However, we note that the aim of this study was to gain insight into infant gut microbiome compositions as they exist among the population and characterize the functional capacities of these communities, rather than to identify the relative impact of medically necessary interventions on the gut microbiome of infants. Further, associations between these interventions and the gut microbiome may be context-dependent, and connections between cesarean section delivery on the infant gut microbiome in the US do not match observations in Bangladesh^11,20^.

In conclusion, our survey highlights the microbiome composition and its metabolic capacity among infants in the US. Given recent findings linking the microbiome in early life to key elements of infant health and the understanding of this community has improved, our findings reveal that infants in the US have microbiomes that may fail to provide functions necessary in early life including shaping the immune system, protecting against pathogen colonization, and maximizing nutrition from breastmilk (e.g. HMOs). These diminished ecosystem services suggest that these communities could thus be classified as dysbiotic^8^. Future efforts should be directed to determine the cause and extent of these results and confirm relevance more broadly and in specific populations of concern. No matter the cause, these findings demonstrate a need to develop interventions that address the dysbiosis reported here, considering microbiome functional deficiencies and restoring the necessary ecosystem services to infants.

## Methods

### Sample collection

The study was conducted at five pediatrician offices and two breastfeeding support centers across five geographically diverse states (California, Georgia, Oregon, Pennsylvania and South Carolina). The experimental protocol was approved by Advarra Central IRB (IRB Registration number: 00000971). All methods were carried out in accordance with relevant guidelines and regulations set forth by the Declaration of Helsinki. Informed consent was obtained from a parent or legal guardian of all participants in the study. Generally healthy aged 0–6 months infants were enrolled. Participants were excluded if they presented with jaundice at the time of enrollment, were undergoing current antibiotic treatment, or were diagnosed with carbohydrate malabsorption syndrome. Site personnel processed fresh diapers within eight hours of receipt. If they could not be processed within 8 h, diapers were frozen at − 20 °C for up to 72 h and thawed for 1 h prior to processing. Site personnel collected stool swabs from each diaper and placed them in a barcoded collection tube containing DNA/RNA Shield stabilization buffer (Zymo Research, Irvine CA) and lysis beads. Stool swab samples in stabilization buffer were stored at room temperature. Additionally, staffs were instructed to aliquot at least a bean-sized amount of stool into an empty, barcoded specimen tube. This aliquot was stored at − 20 °C prior to shipment. All samples were shipped on dry ice to Evolve BioSystems, Inc. Upon receipt, all samples were stored at − 80 °C until further processing. 229 stool samples were collected during the enrollment period but only 227 passed final quality filtering.

### DNA extraction and metagenomics sequencing

gDNA was extracted from 229 stool swab samples stored in DNA/RNA shield lysis tubes (Zymo Research, Irvine CA) using the ZymoBIOMICS 96 MagBead DNA kit (Zymo Research, Irvine CA). Extracted DNA was quantified using QuantIT dsDNA Assay kit, high sensitivity (ThermoFisher Scientific, Waltham, MA) according to the manufacturer’s protocol. 2 Samples did not meet library preparation input specifications and were omitted from downstream analysis. Libraries were prepared for each sample using the Illumina Nextera DNA Flex library kit (Illumina, San Diego, CA) with unique dual indexes according to manufacturer guidelines. Libraries were pooled and submitted to UC Davis DNA Technologies core for sequencing on the Illumina NovaSeq S4 flow cell. (Illumina, San Diego, CA). Each lane of the S4 flow cell contained 96 libraries.

Total *Bifidobacterium* was quantified using a standard curve Taqman-based quantitative PCR assay as previously described using the Bif F, Bif R and Bif P primer/probe set^31,60^. The primer final concentrations were adjusted to 900 nM. Quantification of the total *B. infantis* was performed by quantitative real-time PCR using Blon_2348 sialidase gene primers Inf2348F (5′-ATA CAG CAG AAC CTT GGC CT-3′), Inf2348_R (5′-GCG ATC ACA TGG ACG AGA AC-3′), and Inf2348_P (5′-/56-FAM/TTT CAC GGA /ZEN/TCA CCG GAC CAT ACG/3lABkFQ/-3′)^61^. Each reaction contained 10 μL of 2 × TaqMan Universal Master Mix II with UNG master mix (ThermoFisher Scientific, Waltham, MA), 0.9 µM of each primer, 0.25 µM probe and 5 μL of template DNA. Thermal cycling was performed on a QuantStudio 3 Real-Time PCR System and consisted of an initial UNG activation step of 2 min at 50 °C followed by a 10 min denaturation at 95 °C, succeeded by 40 cycles of 15 s at 95 °C and 1 min at 60 °C. Standard curves for absolute quantification were generated using genomic DNA extracted from a pure culture of *B. infantis* EVC001.

### Quality filtering and removal of human sequences

Quality filtering and removal of human sequences were performed following a previously described pipeline^29^. Demultiplexed Fastq files from NovaSeq were quality filtered using Trimmomatic v0.36^62^ with default parameters. Quality-filtered sequences were screened to remove human sequences using GenCoF v1.0^63^ against a non-redundant version of the Genome Reference Consortium Human Build 38, patch release 7 (GRCh38_p7; www.ncbi.nlm.nih.gov). Human sequence-filtered raw reads were deposited in the Sequence Read Archive (SRA; https://www.ncbi.nlm.nih.gov/sra) under the reference number PRJNA633576.

### Taxonomic and strain profiling

Taxonomic and strain profiling was performed as previously described^29^. Taxonomic profiling of the metagenomic samples was performed using MetaPhlAn2^64^, which uses a library of clade-specific markers to provide pan microbial (bacterial, archaeal, viral, and eukaryotic) profiling (http://hutten-hower.sph.harvard.edu/metaphlan2), in combination with HUMAnN2^65^ to profile functional metagenomics against Uniref90 following the updated global profiling of the Human Microbiome Project (2017)^66^. Strain-specific gene markers were used to determine the presence of *B. longum* subs. *infantis* as previously described^29^. Cross database annotations (e.g., UniProt to KEGG) were performed within Humman2 using the “utility_mapping” conversion tool package.

### Antibiotic resistance gene analysis

Antibiotic resistance gene analysis was computed as previously described^29^. We applied ShortBRED^67,68^ to profile antibiotic resistance (AR) abundance and composition in the infant gut microbiome. We first produced a set of new AR marker sequences by applying ShortBRED-Identify to the Comprehensive Antibiotic Resistance Database database (CARD)^69^. We then used ShortBRED-Quantify to profile the relative abundance of corresponding antibiotic resistance genes (ARGs). Final values were normalized in Reads Per Kilobase per Million mapped reads (RPKM) to account for sequencing depth as well as gene length. We used custom scripts to collapse CARD individual antibiotic resistance gene entries in their corresponding drug class. Conversion rules are offered within every CARD package update.

### Statistical analysis

All statistical analyses were performed in R v3.6.2. A Kruksal-Wallis one-way analysis of variance coupled with an FDR or Bonferroni correction was used for statistical comparisons between individual genes and taxa among groups. Statistical analysis to assess total resistome or enterotype composition by group was performed using a Mann–Whitney or Holm–adjusted Dunn's test. Rarefaction curves were computed to estimate the diversity of the identified ARGs across samples. A nonparametric two-sample t-test was used to compare rarefaction curves using Monte Carlo permutations (*n* = 999). The microbiome multivariable association analysis with linear models was perfomed using MaAsLin2 (https://huttenhower.sph.harvard.edu/maaslin/), by including all the clinical variables presented in the Supplementary Table 1 to test for microbiome differences at the family level according to metadata. All variables were run as fixed effects, except for the variable “State”, which was run as a random effect for the model (Supplementary Table 1). Enterotype analysis was performed as previously described^70^. Principal component analysis and Bray–Curtis beta diversity to confirm enterotype classification was performed as previously decribed^29^, and confirmed with a permutational multivariate analysis of variance (adonis) to test whether the clustering by our Enterotype classification was supported. The *P*-values throughout the manuscript are represented by asterisks (*P < 0.05; **P < 0.01; ***P < 0.001; ****P < 0.0001).

## Supplementary Information

Supplementary Figure 1.

Supplementary Figure 2.

Supplementary Figure 3.

Supplementary Figure 4.

Supplementary Figure 5.

Supplementary Table 1.

Supplementary Table 2.

Supplementary Table 3.

Supplementary Table 4.

Supplementary Table 5.

Supplementary Table 6.

Supplementary Table Legend.

Supplementary Figure Legend.
