# Author Correction: Metagenomic insights of the infant microbiome community structure and function across multiple sites in the United States

**DOI:** 10.1038/s41598-021-90391-4

**Published:** 2021-05-20

**Authors:** Giorgio Casaburi, Rebbeca M. Duar, Heather Brown, Ryan D. Mitchell, Sufyan Kazi, Stephanie Chew, Orla Cagney, Robin L. Flannery, Karl G. Sylvester, Steven A. Frese, Bethany M. Henrick, Samara L. Freeman

**Affiliations:** 1 Evolve BioSystems, Inc., Davis, CA 95618 USA; 2 Department of Surgery, Stanford University, Stanford, CA USA; 3 Department of Food Science and Technology, University of Nebraska, Lincoln, NE 68588 USA; 4 Present Address: Department of Nutrition, University of Nevada, Reno, Reno, NV 89557 USA

Correction to: *Scientific Reports* 10.1038/s41598-020-80583-9, published online 21 January 2021

In this Article the Competing Interests section was incomplete.

“KGS is a professor at Stanford University. GC, RMD, HB, RDM, SK, SC, OC, RLF, SAF, BMH and SLF are employees of Evolve BioSystems, a company focused on restoring the infant microbiome. We are committed to making our raw data, materials, and analysis methods publicly available.”

should read:

“KGS is a professor at Stanford University. SAF and BMH serve as Adjunct Assistant Professors at the Food Science and Technology Department at the University of Nebraska, Lincoln. GC, RMD, HB, RDM, SK, SC, OC, RLF, SAF, BMH and SLF are employees of Evolve Biosystems, a company focused on restoring the infant microbiome that markets a food for special dietary use containing *Bifidobacterium longum* subspecies *infantis*.”

A Data Availability section was originally not included—it should appear as below:

**Data availability**

“The human sequence-filtered raw readers are available at the Sequence Read Archive (SRA; https://www.ncbi.nlm.nih.gov/sra), under the reference number PRJNA633576.”

