# Supplementary figures and images for: Metagenomic insights of the infant microbiome community structure and function across multiple sites in the United States
Source: Sci Rep. 2021 Jan 21;11:1472. doi: 10.1038/s41598-020-80583-9 (PMC7820601; doi:10.1038/s41598-020-80583-9)

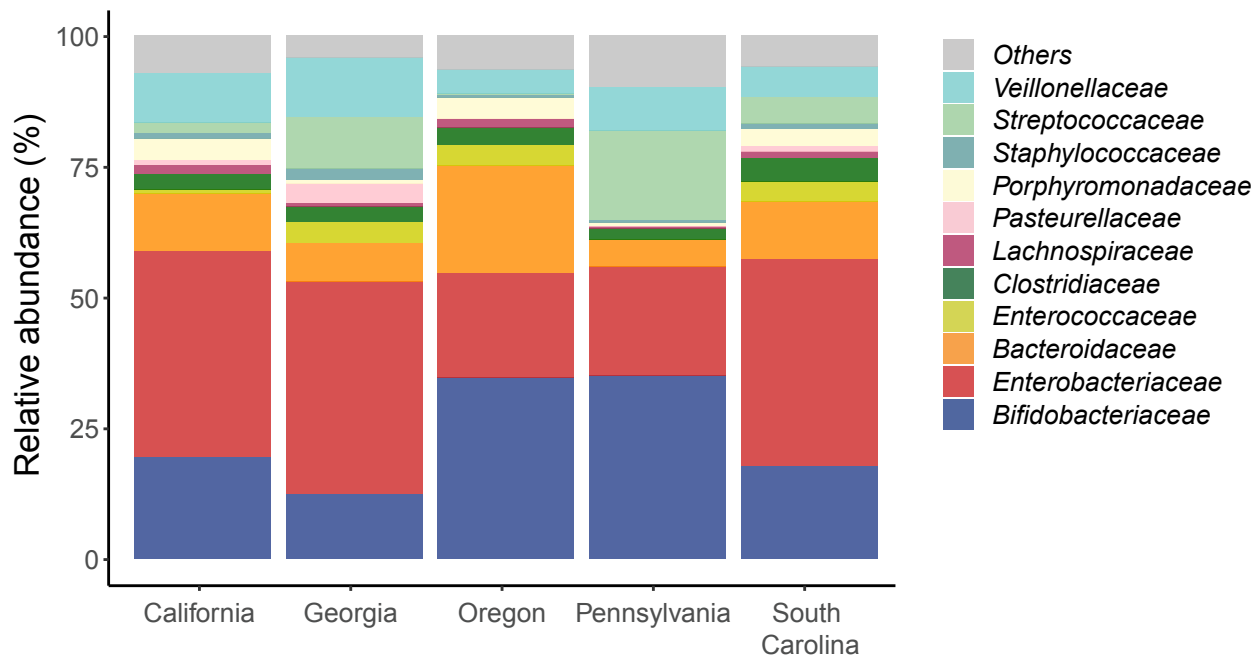

Supplement: Supplementary file 1 — Supplementary Figure 1. [file 41598_2020_80583_MOESM1_ESM.pdf]

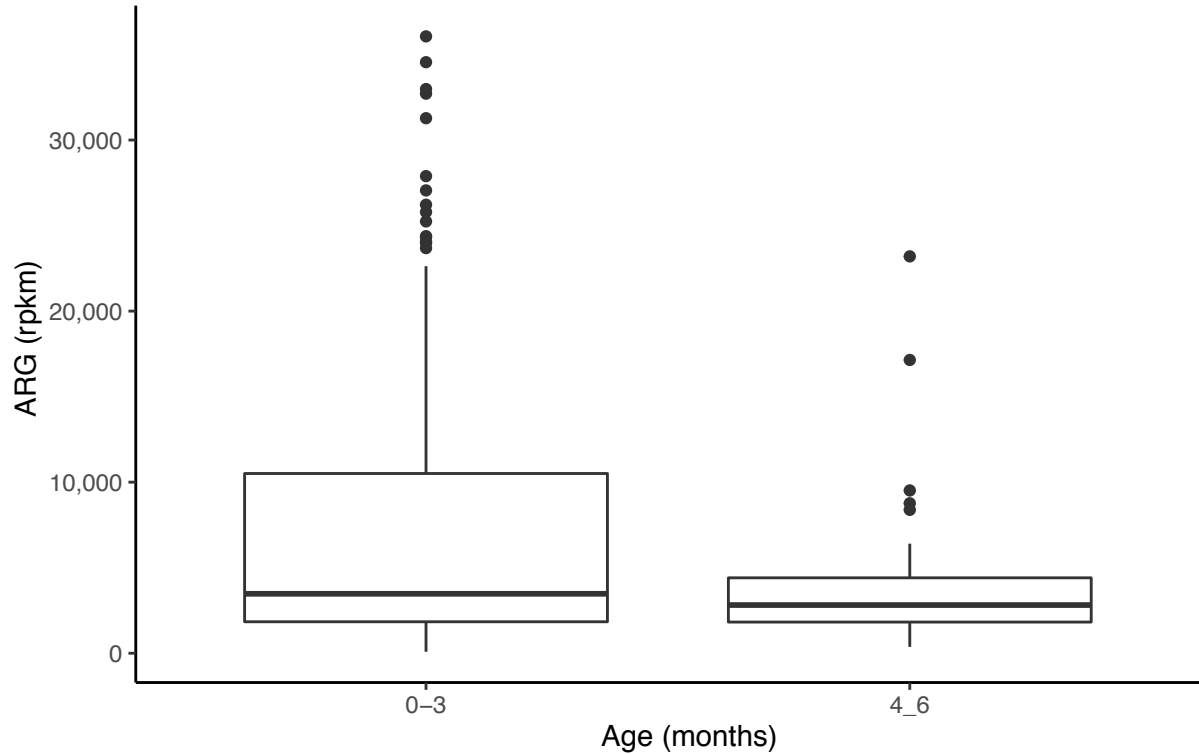

Supplement: Supplementary file 2 — Supplementary Figure 2. [file 41598_2020_80583_MOESM2_ESM.pdf]

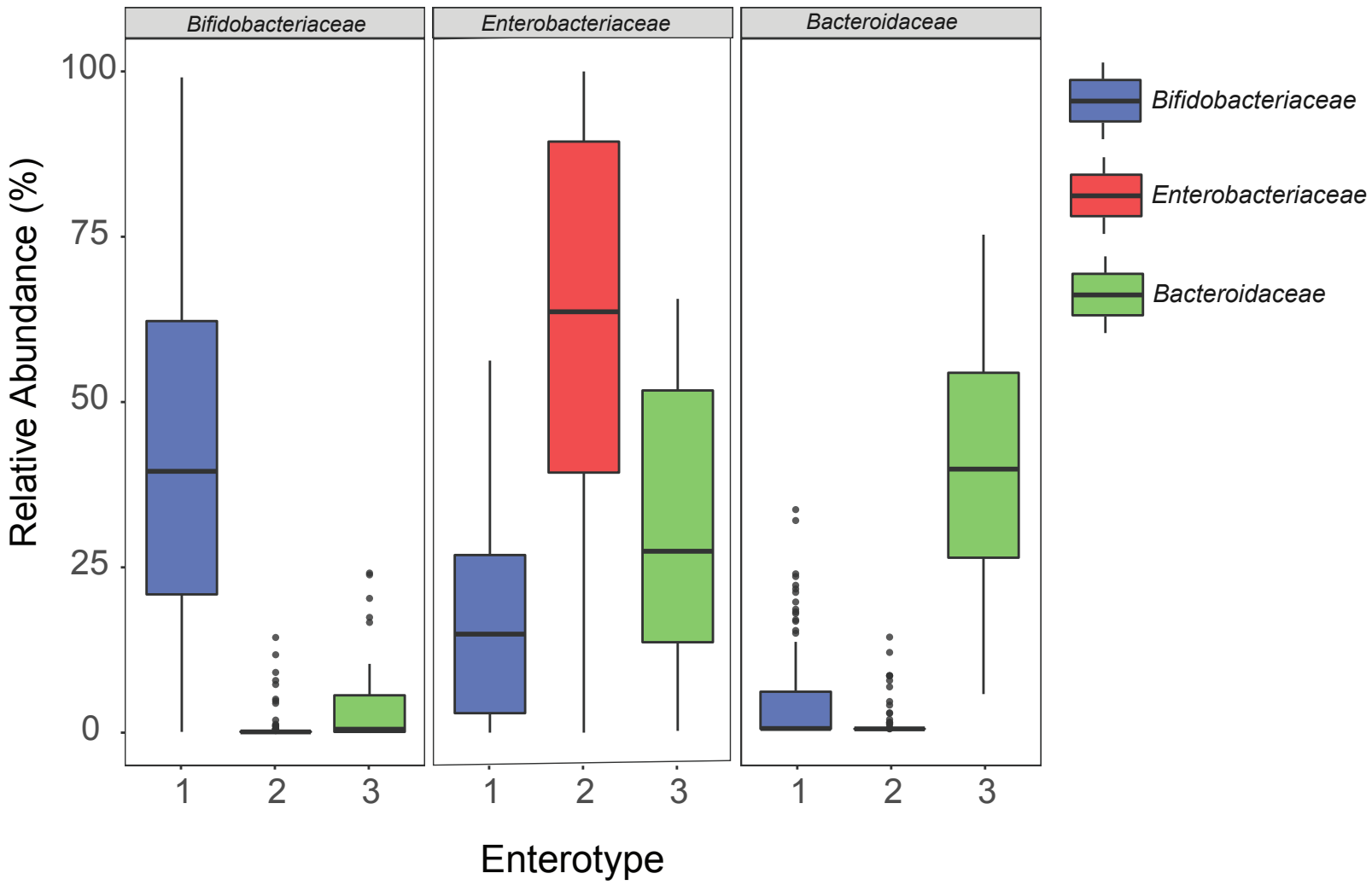

Supplement: Supplementary file 4 — Supplementary Figure 4. [file 41598_2020_80583_MOESM4_ESM.pdf]

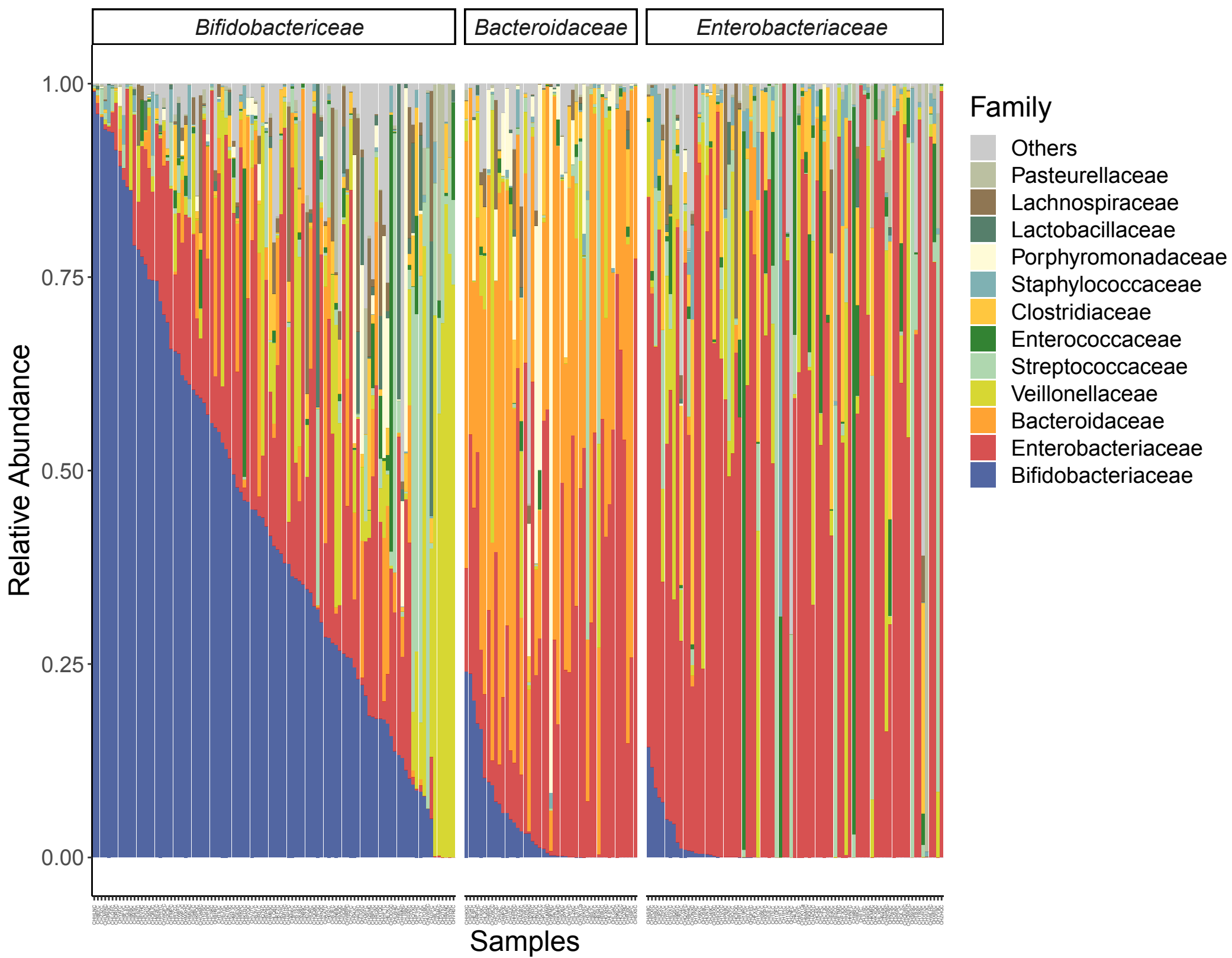

Supplement: Supplementary file 5 — Supplementary Figure 5. [file 41598_2020_80583_MOESM5_ESM.pdf]
