# Supplementary material for: Metagenomic insights of the infant microbiome community structure and function across multiple sites in the United States
Source: Sci Rep. 2021 Jan 21;11:1472. doi: 10.1038/s41598-020-80583-9 (PMC7820601; doi:10.1038/s41598-020-80583-9)
Supplement: Supplementary file 3 — Supplementary Figure 3. [file 41598_2020_80583_MOESM3_ESM.pdf]

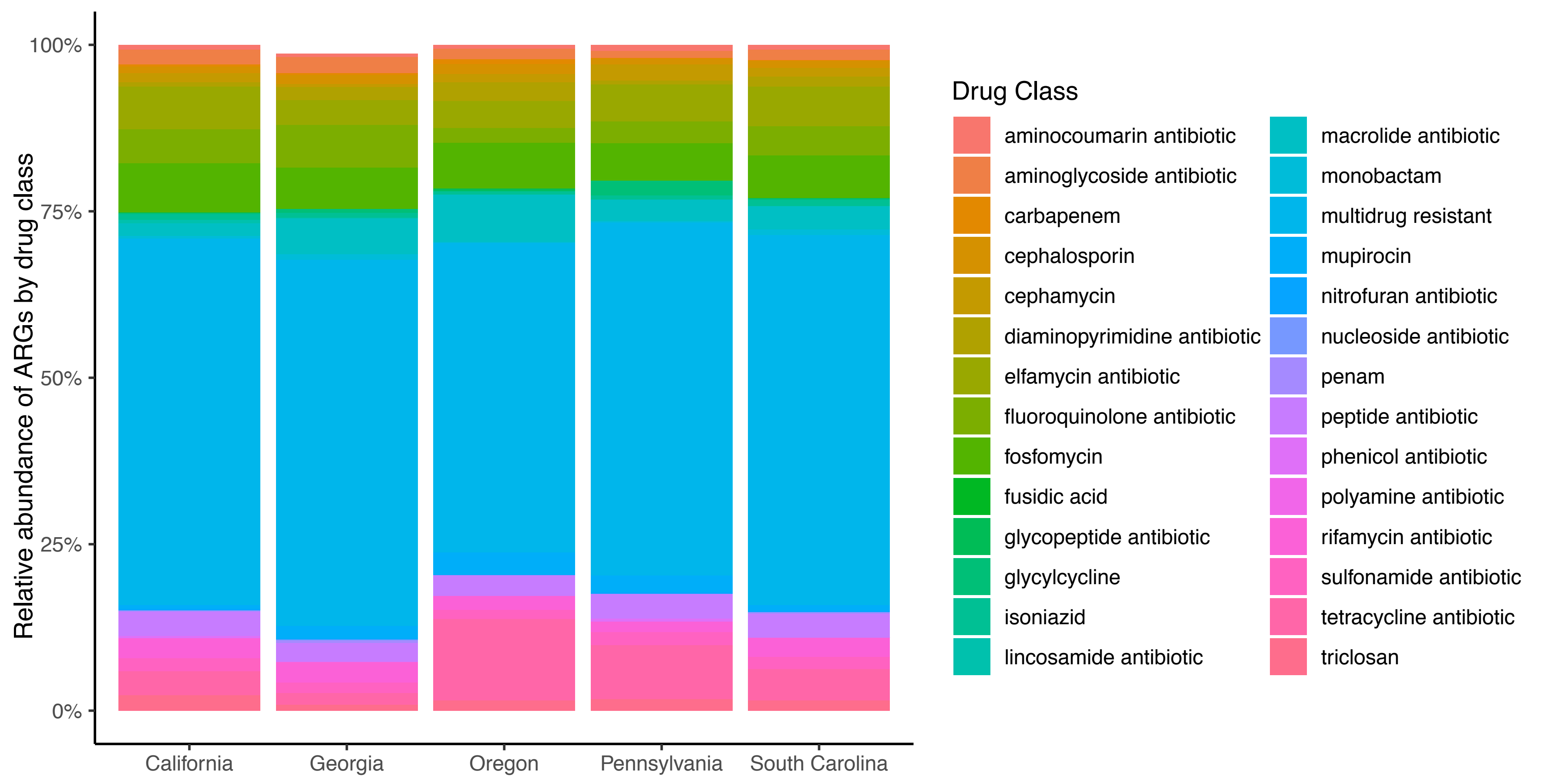
Relative abundance of ARGs by drug class
100%
75%
50%
25%
0%
California
Georgia
Oregon
Pennsylvania
South Carolina
Drug Class
aminocoumarin antibiotic
aminoglycoside antibiotic
carbapenem
cephalosporin
cephamycin
diaminopyrimidine antibiotic
elfamycin antibiotic
fluoroquinolone antibiotic
fosfomycin
fusidic acid
glycopeptide antibiotic
glycylcycline
isoniazid
lincosamide antibiotic
macrolide antibiotic
monobactam
multidrug resistant
mupirocin
nitrofuran antibiotic
nucleoside antibiotic
penam
peptide antibiotic
phenicol antibiotic
polyamine antibiotic
rifamycin antibiotic
sulfonamide antibiotic
tetracycline antibiotic
triclosan
