# Supplementary material for: Metagenomic insights of the infant microbiome community structure and function across multiple sites in the United States
Source: Sci Rep. 2021 Jan 21;11:1472. doi: 10.1038/s41598-020-80583-9 (PMC7820601; doi:10.1038/s41598-020-80583-9)
Supplement: Supplementary file 12 — Supplementary Table Legend. [file 41598_2020_80583_MOESM12_ESM.docx]

**Metagenomic insights of the infant microbiome community structure and function across multiple sites in the United States**

Giorgio Casaburi^1*^, Rebbeca M. Duar^1^, Heather Brown^1^, Ryan D. Mitchell^1^, Sufyan Kazi^1^, Stephanie Chew^1^, Orla Cagney^1^, Robin L. Flannery^1^, Karl G. Sylvester^2^, Steven A. Frese^1,3^, Bethany M. Henrick^1,3^, Samara L. Freeman^1^

^1^Evolve BioSystems, Inc., Davis, CA 95618, USA.

^2^Department of Surgery, Stanford University, Stanford, California, United States of America.

^3^Department of Food Science and Technology, University of Nebraska, Lincoln, NE 68588, USA.

*Corresponding author email: gcasaburi@evolvebiosystems.com

**Supplemental Table Legend:**

**Supplemental Table 1.** Demographic data for every samples collected in this study.

**Supplemental Table 2.** Complete taxonomic profile for every sample in the survey. Taxa are stratifed and reported in relative abundance (%). Same taxonomic ranks sum to 100% [e.g., K__(Kingdom);p__(Phylum);c__(Class);o__(Order);f__(Family);g__(Genus);s__(Species);t__(Strain)]

**Supplemental Table 3.** Antibiotic resistance genes (ARGs) identified by sample and expressed in RPKM.

**Supplemental Table 4.** Antibiotic resistance genes (ARGs) identified by state and expressed in mean RPKM.

**Supplemental Table 5.** Adonis analysis of resitome composition for individual ARG or their respective drug class by different demographic variables. Effect-size (R2) is reported to assess the strength of individual demographic variables to the overall resistome variability (R2: 0-0.2 = weak | 0.2-0.4 = mild | 0.4-0.6 = moderate | 0.6-0.8 = moderately strong | 0.8-1 = strong.

**Supplemental Table 6.** Results of the MaAsLin2 model showing effects of individual covariates on the bacterial families’ composition in terms of coefficient and statistical significance. Total average of bacterial families is also reported for context.
