# Supplementary material for: Metagenomic insights of the infant microbiome community structure and function across multiple sites in the United States
Source: Sci Rep. 2021 Jan 21;11:1472. doi: 10.1038/s41598-020-80583-9 (PMC7820601; doi:10.1038/s41598-020-80583-9)
Supplement: Supplementary file 13 — Supplementary Figure Legend. [file 41598_2020_80583_MOESM13_ESM.docx]

**Metagenomic insights of the infant microbiome community structure and function across multiple sites in the United States**

Giorgio Casaburi^1*^, Rebbeca M. Duar^1^, Heather Brown^1^, Ryan D. Mitchell^1^, Sufyan Kazi^1^, Stephanie Chew^1^, Orla Cagney^1^, Robin L. Flannery^1^, Karl G. Sylvester^2^, Steven A. Frese^1,3^, Bethany M. Henrick^1,3^, Samara L. Freeman^1^

^1^Evolve BioSystems, Inc., Davis, CA 95618, USA.

^2^Department of Surgery, Stanford University, Stanford, California, United States of America.

^3^Department of Food Science and Technology, University of Nebraska, Lincoln, NE 68588, USA.

*Corresponding author email: gcasaburi@evolvebiosystems.com

**Supplemental Figure Legend:**

**Supplemental Figure 1.** Relative abundance (%) of top bacterial families identified in infants 0-3 months of age across five US states.

**Supplemental Figure 2.** Comparison of the total load of ARGs (RPKM) in the microbiome of infants between 0-3 and 4-6 months of age (*P* > 0.05).

**Supplemental Figure 3.** Relative abundance (%) of ARGs drug class compared by state.

**Supplemental Figure 4.** Relative abundance (%) of the three main bacterial families driving the three enterotypes. Samples were grouped based on the enterotype they belong to (bottom numbers).

**Supplemental Figure 5.** Relative abundance (%) of top bacterial families with at least 1% in relative abundance on average and showed on a per sample basis and sorted by Enterotype.
